# An Investigation on the Synthesis of Molybdenum Oxide and Its Silica Nanoparticle Composites for Dye Degradation

**DOI:** 10.3390/nano10122409

**Published:** 2020-12-02

**Authors:** Olfa Kamoun, Abdelaziz Gassoumi, Salah Kouass, Badriyah Alhalaili, Ruxandra Vidu, Najoua Turki-Kamoun

**Affiliations:** 1 Laboratoire de Physique de la Matière Condensée, Faculté des Sciences de Tunis, Université de Tunis El Manar, Tunis 2092, Tunisia; o.kamoun@yahoo.fr (O.K.); n.najouakamoun@gmail.com (N.T.-K.); 2 Department of Physics, Faculty of Science, King Khalid University, P.O. Box 9004, Abha 61413, Saudi Arabia; 3 Laboratoire Matériaux Utiles, Institut National de Recherche et d’Analyse Physico-Chimique (INRAP) Sidi Thabet, Ariana 2020, Tunisia; kouasssa@gmail.com; 4 Nanotechnology and Advanced Materials Program, Kuwait Institute for Scientific Research, P.O. Box 24885, Safat 13109, Kuwait; bhalaili@kisr.edu.kw; 5 Department of Electrical and Computer Engineering, University of California Davis, Davis, CA 95616, USA; 6 Faculty of Materials Science and Engineering, University POLITEHNICA of Bucharest, 313 Splaiul Independentei, RO-060042 Bucharest, Romania

**Keywords:** MoO_3_ nanoparticles, silica, nanoparticle composite, structural properties, photocatalytic properties, methylene blue

## Abstract

The molybdenum oxide (MoO_3_) and MoO_3_@SiO_2_ nanoparticles were successfully prepared using the chemical bath deposition (CBD) method. The photocatalytic activities of molybdenum oxide (MoO_3_), SiO_2_, and MoO_3_@SiO_2_ nanoparticles composite have shown a synergistic photocatalytic effect of SiO_2_ combined with MoO_3_. The first-order degradation rate constants for MoO_3_, SiO_2_, and MoO_3_@SiO_2_ nanocomposite were 10.3 × 10^−3^ min^−1^, 15.1 × 10^−3^ min^−1^, and 16.3 × 10^−3^ min^−1^, respectively. The MoO_3_@SiO_2_ composite showed degradation efficiencies in the methylene blue solution close to 100% after 60 min of UV irradiation. The X-ray diffraction (XRD) showed that the MoO_3_ powder has a hexagonal crystal structure and the silica is the tridymite type of SiO_2_. The crystallite size was about 94 nm, 32 nm, and 125 nm for MoO_3_, silica, and MoO_3_@SiO_2_, respectively, as calculated by the Scherrer equation. The scanning electron microscopy (SEM) images revealed that the MoO_3_ powder consisted of a uniform hexagonal structure; the silica showed a rod-like micro-flake morphology and the MoO_3_@SiO_2_ composite had the appearance of coral-like structures.

## 1. Introduction

Catalytic materials were developed over the years to purify the polluted water and air, where the heterogeneous photocatalysis plays an important role [1,2,3,4,5]. The well-known transition metal oxide photocatalysts are ZnO [6], TiO_2_ [7], WO_3_ [8], and MoO_3_ [9]. The oxidation process of heterogeneous photocatalysis is achieved by using light to activate the catalyst and to generate highly reactive free radicals, which then reduce particular organic compounds [6,7,9,10,11]. When the mineralization process is finished, the outcome consists of H_2_O and CO_2_.

The photocatalytic mechanism is activated when the oxide semiconductor is immersed in a liquid or placed in a gaseous medium and then irradiated with light of an energy that is equal to or greater than its bandgap [11]. In this case, electron-hole pairs are generated on the surface of the semiconductor and subsequent chemical reactions with the environmental media lead to the production of free radicals, which degrade the organic pollutants [10,11].

Photocatalysis requires a large surface area for reaction. The exterior of natural silica is covered with a network of pores to optimize the capture of light. This feature of silica has attracted the attention of nanotechnologists. Moreover, silica is a phylum of unicellular microalgae (from 2 μm to 1 mm) present in all aquatic environments, and the majorities are in biofilms (with a preference for cold water) and enveloped by a siliceous external skeleton. The degradation of MB under the visible light has been demonstrated for photocatalysts prepared using green and renewable resources [12]. Mesoporous silica impregnated with Pt-Porphyrin or PtNPs [13] and titania sensitized with porphyrin [14] or magnetic photocatalyst porphyrin [15] has also been shown visible-light-driven photocatalysis. MoO_3_ is one of the most promising metal oxides because of its many advantages, such as its non-toxic nature, and can be widely used in an organic light-emitting diode, gas sensing, catalysis, transistors, and solar cells [16,17,18]. The smaller the particle size of MoO_3_, the larger the surface area, which potentially increases the adsorption methylene blue and photoactive sites, resulting in enhanced photocatalytic activity [9,16,19]. Various methods have been used to optimize the preparation and processing technology of molybdenum oxide films, such as thermal evaporation, sol–gel deposition, and chemical vapor deposition [20,21,22]. In our work, we used a simple and inexpensive chemical bath deposition (CBD) technique because the film properties can be optimized through various deposition process parameters. The CBD technique has received great consideration from the research community for the production of low-cost semiconductor photocatalyst.

In our search for novel and sustainable photocatalysts, we used the unique architecture of silica and high surface area to increase the degradation efficiency of the organic dyes. Because MoO_3_ has shown good photocatalytic degradation properties [23,24,25], present in this study the synthesis of a novel MoO_3_ and SiO_2_ nanoparticle composites, labeled MoO_3_@SiO_2_, and their photocatalytic properties. The main objective of this work is to study the photocatalytic activities of the films for the photodegradation of methylene blue (MB) under UV light, as well as examine the physical properties of the MoO_3_ films.

## 2. Experimental and Characterization Details

**Figure 1:**
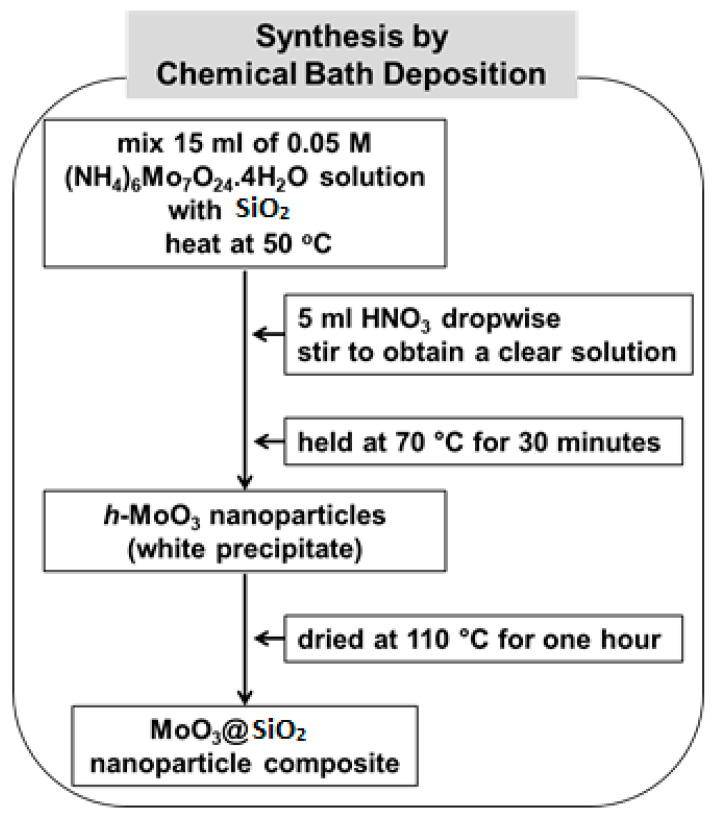
Illustration of the processing steps in the synthesis of MoO_3_@SiO_2_ nanoparticle composites.

The chemical bath deposition (CBD) method was performed to synthesize nanocrystalline MoO_3_ and MoO_3_ on SiO_2_, as illustrated in Figure 1. In a typical synthesis, an aqueous solution of 15 mL of 0.05 M (NH_4_)_6_Mo_7_O_24_·4H_2_O (99%, Merck, Kenilworth, NJ, USA) solution was mixed with SiO_2_ in a reaction bath. The temperature of the reaction bath was slowly increased to 50 °C. Then, 5 mL of concentrated HNO_3_ (ACS reagent, ≥90.0%) was added dropwise with constant stirring until the pH of the solution was 2.2, and a clear solution was obtained. Then, after the solution was stirred for 15 min, the temperature of the reaction bath was raised to 70 °C, where the initial seeds started to form. The reaction bath was held at 70 °C for 30 min, during which time a white precipitate of h-MoO_3_ nanoparticles was observed. When the synthesis was complete, the white precipitate was filtered using deionized water and then dried in an oven at a constant temperature of 110 °C for 1 h [26,27]. 

The nanoparticle composites were analyzed by X-ray diffraction (XRD) scanning electron microscopy (SEM), and UV-Vis spectroscopy. The XRD patterns were recorded on an X’pert PRO X-ray diffractometer (Malvern Panalytical Ltd, Malvern, UK) with graphite monochromatized Cu Kα radiation source (1.5406 Å). Morphologies of nanopowders were examined using a JEOL-JSM-6490 LV scanning electron microscope (SEMTech Solutions, Inc., North Billerica, MA, USA) and absorbance measurements were performed using a Perkin Elmer Lambda 950 spectrometer (Perkin Elmer, Waltham, MA, USA). The UV irradiation was performed at 254 nm wavelength using an 8 W power lamp (Philips Germicidal Ultraviolet-C, Philips Lightning, Eindhoven, The Netherlands).

## 3. Results and Discussion

### 3.1. Structural Properties

**Figure 2:**
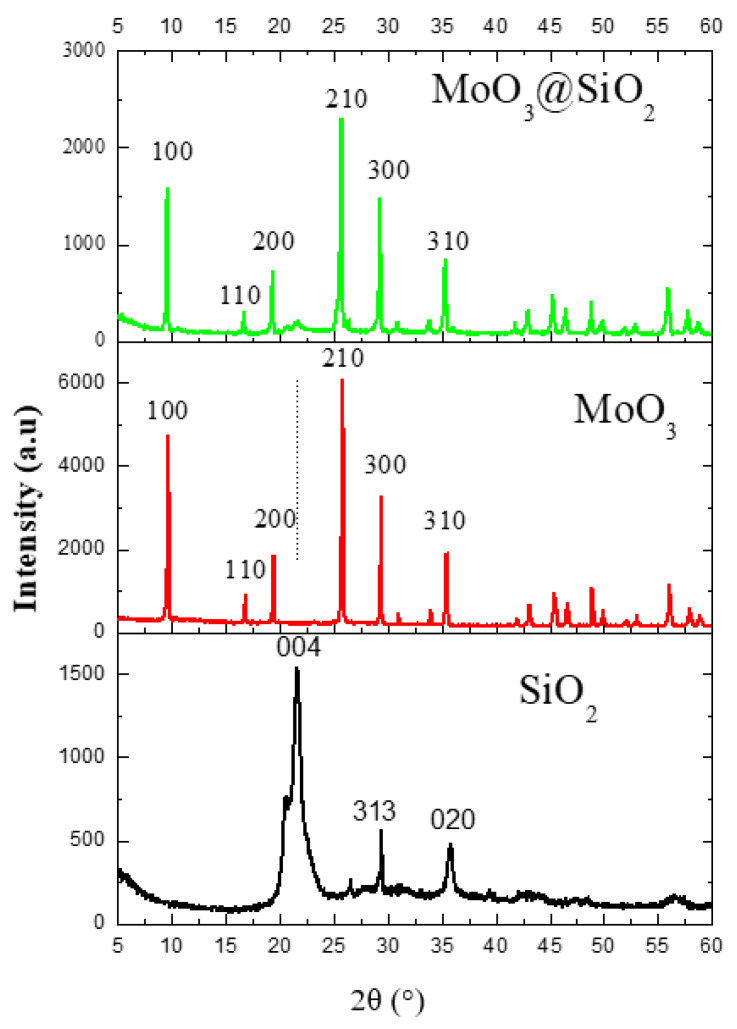
The XRD patterns for the SiO_2_, MoO_3,_ and the MoO_3_@SiO_2_ composite.

Both MoO_3_ nanoparticles and SiO_2_ were analyzed by X-ray diffraction analysis and compared to the MoO_3_@SiO_2_ nanoparticles composite. Figure 2 shows the XRD patterns of the MoO_3_, SiO_2,_ and MoO_3_@SiO_2_ nanoparticles grown by chemical bath deposition. The diffraction patterns correspond to the h-MoO_3_ phase for the MoO_3_ nanoparticles and to tridymite, which is the monoclinic phase corresponding to SiO_2_ for the silica [28]. 

Tridymite is a species of mineral of the tectosilicate family, and one of the polymorphs of silica with quartz, coesite, cristobalite, stishovite, having the chemical formula of SiO_2_ and containing traces of titanium, aluminum, iron, manganese, magnesium, calcium, sodium, and potassium.

MoO_3_ has the following lattice parameters: a = 10.53 Å and c = 14.876 Å (JCPDS card no. 21-0569) [19]. The SiO_2_ has the following lattice parameters: a = 25.93 Å, b = 5.01 Å, and c = 18.54 Å, with the highest intensity at 2θ = 21.6°, matching the reference R090042 [29]. 

The crystallite size can be estimated from the full width half maximum (FWHM) values obtained from the predominant (210) for MoO_3_ and MoO_3_@SiO_2_ diffraction peak at 2θ = 25.7° according to the following Debye–Sherrer equation [30,31,32]:

D = 0.9λ/βcosθ(1)

where λ is the wavelength of Cu-Kα1 radiation (1.5406 Å) and θ is the Bragg diffraction angle. The crystallite sizes calculated with Equation (1) were around 94, 32, and 125 nm for MoO_3_, SiO_2,_ and MoO_3_@SiO_2_, respectively. The observed broadening of the SiO_2_ peak(004) in the MoO_3_@SiO_2_ spectrum is attributed to the size and strain effect between MoO_3_ and SiO_2_ [19].

In the XRD spectra of the MoO_3_@SiO_2_ composite, all peaks attributed to the MoO_3_phase are observed, which confirms that the MoO_3_ nanoparticles are well grown on the SiO_2_ surface. It was also observed that the intensity for all MoO_3_ peaks decreases for MoO_3_@SiO_2_ composite compared to those of MoO_3_. Note the existence of the preferential SiO_2_ peak at 2θ = 21.6°. All MoO_3_@SiO_2_ peaks decreased in intensity, which may be due to the fact that MoO_3_ nanoparticles are well-formed on the surface of SiO_2_, but in a dispersed manner.

### 3.2. Morphological Analysis

**Figure 3:**
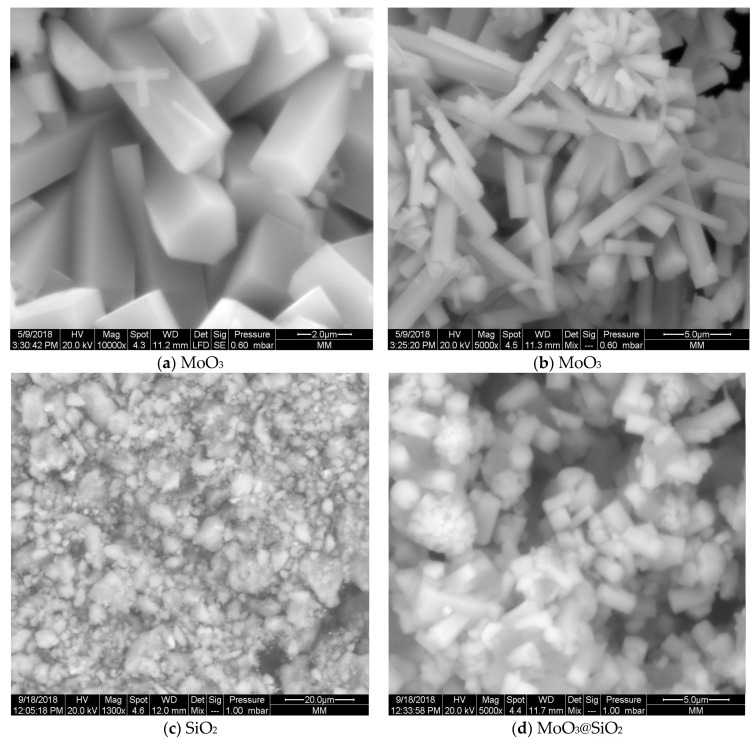

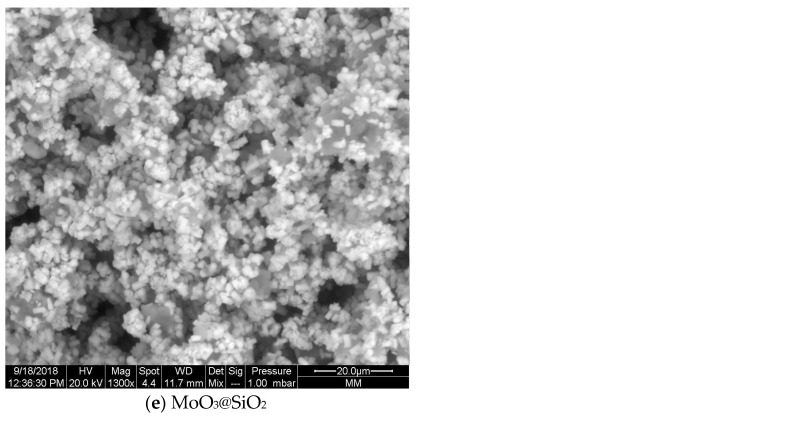
SEM micrographs of MoO_3_nanoparticles (**a**,**b**), silica particles (**c**), and MoO_3_@SiO_2_ nanoparticles composites (**d**,**e**) at different magnifications.

Surface morphology of the nanoparticles composite and constituent nanoparticles were investigated by using SEM analyses. The nanoparticles of MoO_3_ consist of a uniform hexagonal rod-like morphology. The regular faceted surface of each hexagonal rod [33] is clearly seen in Figure 3a. In Figure 3b, stems have developed out of a central point [34], with flower-like clusters of hexagonal MoO_3_ stem-shaped petals. The SEM images of SiO_2_ (Figure 3c) shows a similar morphology as SiO_2_ [35]. The morphology of SiO_2_ depicts mostly micro-flake and irregular rod-shaped with particles agglomeration. Regarding the MoO_3_@SiO_2_ composite (Figure 3d,e), we observed the appearance of coral-like structures in the form of hexagonal rods. The morphology of this composite indicates the incorporation of MoO_3_ into the SiO_2_ in the MoO_3_@SiO_2_ composite, which is in agreement with the XRD analysis in Figure 2. SEM observation shows that the specific surface area increased for the MoO_3_@SiO_2_ composite compared to those for MoO_3_ or SiO_2_. Increasing the specific surface area, especially in the case of MoO_3_@SiO_2_, could play an important role in improving sensitivity in optoelectronic applications like photocatalysis and gas sensors.

### 3.3. Photocatalytic Studies

**Figure 4:**
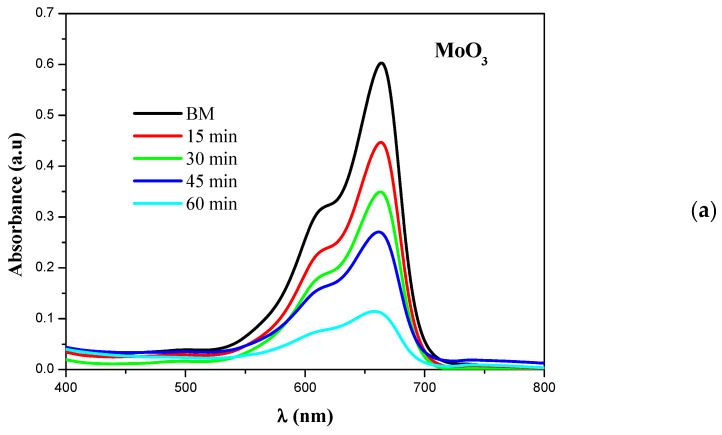

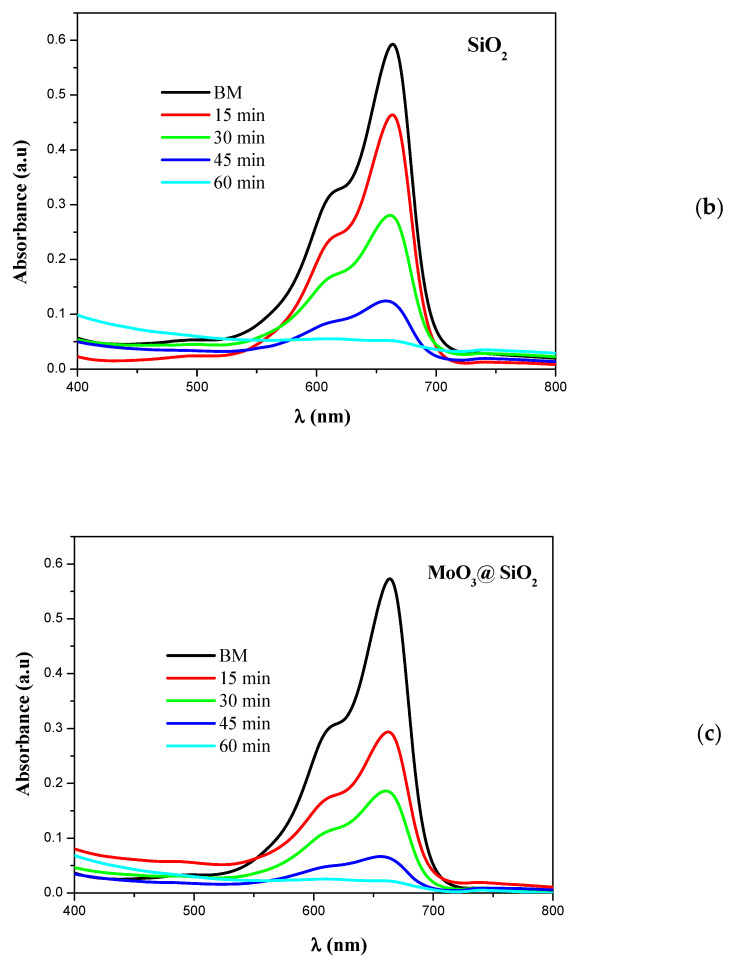
UV-Vis absorption spectra of MoO_3_ nanoparticles (**a**), SiO_2_ (**b**) and MoO_3_@SiO_2_ (**c**).

MoO_3_ nanoparticles were used as photocatalyst for the degradation of methylene blue (MB), which was used as a model compound. It was found that there was no MB degradation in the dark and in the presence of MoO_3_, SiO_2_, and MoO_3_@SiO_2_. In this work, we have monitored the MB degradation under UV light at different times with MoO_3_, SiO_2_, and MoO_3_@SiO_2_ nanoparticle catalysts. Figure 4 presents the UV-Vis absorption spectra of MoO_3_ nanoparticles, SiO_2_ and MoO_3_@SiO_2_ nanoparticles exposed to UV light for different times.

There are two different absorption bands for the aqueous cationic MB dye solution, i.e., at 293 nm (π-π∗) and 664 nm (*n*-π∗) [34]. In this work, the intensities of the absorption peaks at 664 nm decrease with increasing the time of irradiation, compared to the catalyst-free solution. The degradation of the MB solution containing h-MoO_3_ catalyst synthesized by CBD was 90% [34]. 

During photocatalysis, the electrons in the valence band of the oxide semiconductor are excited under UV light radiation and leave holes in the valence band after they jump to the conduction band. The holes combine with H_2_O to produce ∙ H and ∙ OH radicals. In the meanwhile, the electrons in the conduction band scattered towards the adsorbed O_2_ to generate activated ∙ O_2_ [36] with the consequent transformation of the water molecules into ∙ OH radicals. 

The mechanism of photocatalytic degradation for MoO_3_nanoparticles is similar to that of a metal oxide semiconductor [37], as follows:

MoO_3_ (or SiO_2_ or composite) + hν→MoO_3_ (e^−^ + h^+^) (2)

h^+^+ OH^−^→OH^.^ (hydroxide) (3)

e^−^ + O_2_→O_2_^−^ (super oxide anion) (4)

OH^.^ + MB→MB^*^ (intermediate) →CO_2_ + H_2_O (5)

O_2_^−^+ MB→MB^*^ (intermediate) →CO_2_ + H_2_O (6)

These oxidizing species can degrade the MB dye into chemical forms of CO_2_ and H_2_O, which is a better solution to water remediation treatments [36]. If the photocatalytic processes do not take place, the recombination of the (e^−^ + h^+^) pairs happens, and heat is generated in the materials. The photocatalytic activity depends on various factors, including the structure and the dimension of the particles, degree of crystallinity, specific surface area, adsorbed water molecules, and hydroxyl groups [38,39,40,41].

**Figure 5:**
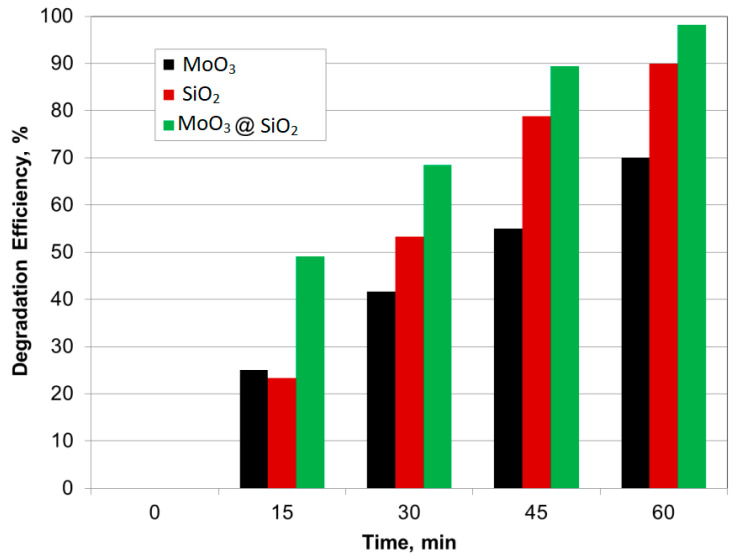
Comparative bar diagram of degradation efficiency for MoO_3_, SiO_2,_ and MoO_3_@SiO_2_.

The degradation efficiency was further studied in the presence of h-MoO_3_, SiO_2_, and MoO_3_@SiO_2_ nanoparticle composite in MB dye, and the results are presented in Figure 5. The degradation efficiency was calculated using the following equation [42,43]:

*Degradation efficiency* = (*C*_0_ − *C*)/*C*_0_ (%) (7)

where *C*_0_ is the initial dye concentration in the solution, and *C* is the dye concentration in the solution after irradiation, for a given time interval [42].

Figure 5 shows that the degradation efficiency increases with exposure time under UV-light. The MoO_3_@SiO_2_ composite showed degradation efficiencies in the MB solution close to 100% after 60 min of UV irradiation. The MoO_3_@SiO_2_ composite showed stable rates of MB photodegradation up to six cycles.

**Figure 6:**
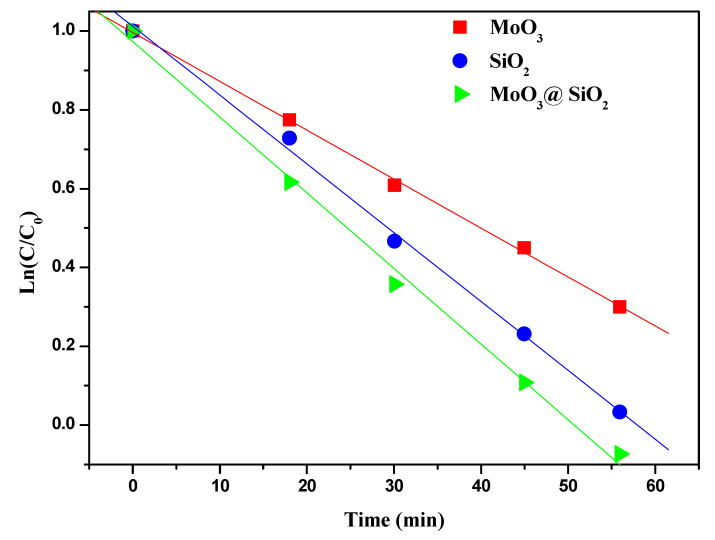
The plots of *ln*(*C*/*C*_0_) versus time for MoO_3_, SiO_2,_ and MoO_3_@SiO_2_.

The rate kinetics analysis, an important parameter in the degradation studies, was performed to predict the rate at which MB is removed from the aqueous solution [42]. In these experiments, different amounts of MoO_3_, SiO_2,_ and MoO_3_@SiO_2_ composite were used with a fixed concentration of MB. The reaction kinetics was calculated with Equation (8) [42]:

*Ln*(*C*/*C*_0_) = −*kt* (8)

where *C*_0_ and *C* were defined for Equation (7). The graph of the natural logarithm, *Ln*(*C*/*C*_0_) for MB dye versus time in the presence of MoO_3_, SiO_2_, and MoO_3_@SiO_2_ nanocomposite is presented in Figure 6.

The MB concentration presented in log scale in Figure 6 varies practically linearly with time, indicating that the photodegradation of MB dye follows the first-order kinetics [42]. The kinetic rate constants (*k*) were determined from the slope of fitted curves. The first-order degradation rate constants for MoO_3_, SiO_2_, and MoO_3_@SiO_2_ nanocomposite were 10.3 × 10^−3^ min^−1^, 15.1 × 10^−3^ min^−1^, and 16.3 × 10^−3^ min^−1^, respectively. Table 1 presents the rate constants for MB degradation obtained in this work in comparison to other literature data. The degradation rate of MB is faster for MoO_3_@SiO_2_ nanocomposite compared to MoO_3_ or SiO_2_.

## 4. Conclusions

We have synthesized MoO_3_@SiO_2_ nanoparticle composite using chemical bath deposition. The diffraction patterns are in good agreement with the hexagonal phase MoO_3_ with the lattice parameters of a = 10.53 Å and c = 14.876 Å. The SiO_2_ has the following lattice parameters: a = 25.93 Å, b = 5.01 Å, and c = 18.54 Å. The XRD analysis showed that MoO_3_, silica, and MoO_3_@SiO_2_ nanoparticle composite have crystalline characteristics phase with an average crystallite size of about 94 nm, 32 nm, and 125 nm, respectively. SiO_2_ showed micro-flakes morphology with agglomeration as confirmed by SEM analysis, irregular rod-shaped for MoO_3_, and coral-like structure for MoO_3_@SiO_2_. The optimum photocatalytic activity was found for MoO_3_@SiO_2_ nanoparticles, with an efficiency of about 100% after 60 min of exposure to the UV-light, while the degradation efficiency for the same UV exposure time was about 90% and 70% for SiO_2_ and MoO_3_, respectively. The degradation rate constants for MoO_3_, SiO_2_, and MoO_3_@SiO_2_ nanocomposite were 10.3 × 10^−3^ min^−1^, 15.1 × 10^−3^ min^−1^, and 16.3 × 10^−3^ min^−1^, respectively. These results show that SiO_2_ particles have a beneficial photocatalytic effect combined with MoO_3_ in the MoO_3_@SiO_2_ composite in the photocatalytic processes.

## Figures and Tables

**Table 1:** Comparative rate constants for different photocatalysts, including our work.

Material	Rate Constants × 10^−3^ min^−1^	References
MoO_3_ (CBD)	10.3	this work
SiO_2_	15.1	this work
MoO_3_@SiO_2_	16.3	this work
MoO_3_	0.334	[44]
ZnO	15.15	[6]
α-Fe_2_O_3_	2.01	[45]
SnS_2_	4.43	[45]
SrFe_12_O_19_	13.6	[46]
TiO_2_	35.58	[47]
